# *ERBB2* Mutations as Potential Predictors for Recurrence in Colorectal Serrated Polyps by Targeted Next-Generation Sequencing

**DOI:** 10.3389/fonc.2022.769709

**Published:** 2022-03-23

**Authors:** Qi-Wen Wang, Xin-Yuan Wang, Qing-Wei Zhang, Jin-Nan Chen, Yu-Jie Zhou, Zhao-Rong Tang, Rui-Lan Wang, Haoyan Chen, Huimin Chen, Xiao-Bo Li

**Affiliations:** ^1^ Division of Gastroenterology and Hepatology, Key Laboratory of Gastroenterology and Hepatology, Ministry of Health, Renji Hospital, School of Medicine, Shanghai Jiao Tong University, Shanghai Institute of Digestive Disease, Shanghai, China; ^2^ Department of Gastroenterology and Hepatology, Chongqing Traditional Chinese Medicine Hospital, Chongqing, China; ^3^ Department of Gastroenterology and Hepatology, Sichuan Provincial Corps Hospital of Chinese People’s Armed Forces, Leshan, China; ^4^ State Key Laboratory for Oncogenes and Related Genes, Shanghai Cancer Institute, Renji Hospital, School of Medicine, Shanghai Jiao Tong University, Shanghai, China

**Keywords:** serrated polyps, serrated pathway, colorectal cancer, *ERBB2*, recurrence

## Abstract

**Background:**

Follow-up guidelines for serrated polyps (SPs) are mainly based on factors such as histology and size with limited evidence. The underlying genomic mechanism of SPs in relation to recurrence risks is utterly unknown.

**Methods:**

We applied targeted next-generation sequencing (NGS) approach on two groups of SPs [polyp-relapsed SPs (PRSPs) vs. polyp-free SPs (PFSPs)] based on the surveillance outcomes to compare differences of DNA variants in 71 colorectal cancer-associated genes. A multicenter validation cohort was established longitudinally from 2016 to 2019 to confirm the relevant results.

**Results:**

Among the 96 NGS samples, at least one mutant after filtration was detected in 90 samples (94%). Molecular profiling presented *BRAF*, *KRAS*, and *APC* as top 3 mutated genes. *FBXW7*, *MSH2*, and *ERBB2* might be recurrence-relevant, while *DMD*, *BRCA1*, and *BRCA2* might be negatively correlated with recurrence. Notably, *ERBB2* mutants (R678Q and V842I) (n = 5) had higher risks of polyp recurrence than the wild types (n = 85), with a median polyp-free interval of 15 months compared to 26 months [P < 0.001; hazard ratio (HR) = 4.9; 95% confidence interval (CI) = 1.9–12.8]. Furthermore, a multicenter cohort composed by 321 SPs verified that *ERBB2*-mutated SPs had increased risks of polyp recurrence (P < 0.001; HR = 3.7; 95% CI = 2.3–6.0) and advanced neoplastic lesion (ANL) recurrence (P < 0.001; HR = 10.0; 95% CI = 2.7–36.9) compared with wild-type SPs, respectively.

**Conclusions:**

Our results are emphasizing that SP individuals with *ERBB2* mutants are at higher risks of subsequent colorectal neoplasms. *ERBB2* mutants might work as facilitated markers for prediction of high-risk SPs and might implicate a potential mechanism in the serrated pathway to colorectal carcinoma (CRC).

## Introduction

Serrated polyps (SPs) are the second most common type of colorectal polyps with a distinct histological appearance of saw-toothed colonic crypts. According to the 2019 World Health Organization’s criteria, SPs are histologically classified as hyperplastic polyps (HPs), sessile serrated lesions (SSLs), or traditional serrated adenomas (TSAs) (1). Unlike the canonical adenoma-carcinoma sequence, SPs are proven to be early precursors to about 15%–30% colorectal carcinoma (CRC) through serrated pathway, frequently associated with *BRAF* or *KRAS* oncogenic mutations, microsatellite instability (MSI), and high CpG island methylator phenotype (CIMP) (2).

There is no consensus in the literature on which SPs are clinically relevant. A wide risk variation exists among SPs, as some case reports present SP rapid progression to invasive cancer within months (3), while other findings suggest a mean interval of 15 years for malignant transformation (4). Several longitudinal studies have investigated the relationship between the future risks of colorectal neoplasms and the clinicopathological characteristics of SPs, such as histology, size, anatomic location, or numbers (5–7). Evidence indicates that the detection of large SPs at the first endoscopy is more likely to have metachronous advanced neoplasms than those with no SPs (8), and it is an independent risk factor for subsequent CRC even with stronger association than that for advanced adenomas (9, 10). Cross-sectional reports have also substantially manifested that over-representation of malignancy hallmarks in SPs including *BRAF* mutation, *MLH1* methylation, *MUC5AC* demethylation, and CIMP proposes possibly higher risks of CRC compared to conventional adenomas (11–13). Nevertheless, little is known about the genomic aberrations of SPs in relation to recurrence risks.

Therefore, we applied targeted next-generation sequencing (NGS) approach on groups of polyp-relapsed SPs (PRSPs) and polyp-free SPs (PFSPs) based on the surveillance outcomes to compare the intergroup differences of variants in 71 colorectal cancer-associated genes. In order to get insight about the value of genomic variants in predicting polyps’ recurrence and in planning colonoscopic surveillance, we further validated our results in a multicenter validation cohort established longitudinally from 2016 to 2019.

## Methods

### Cohort Selection

**Figure 1:**
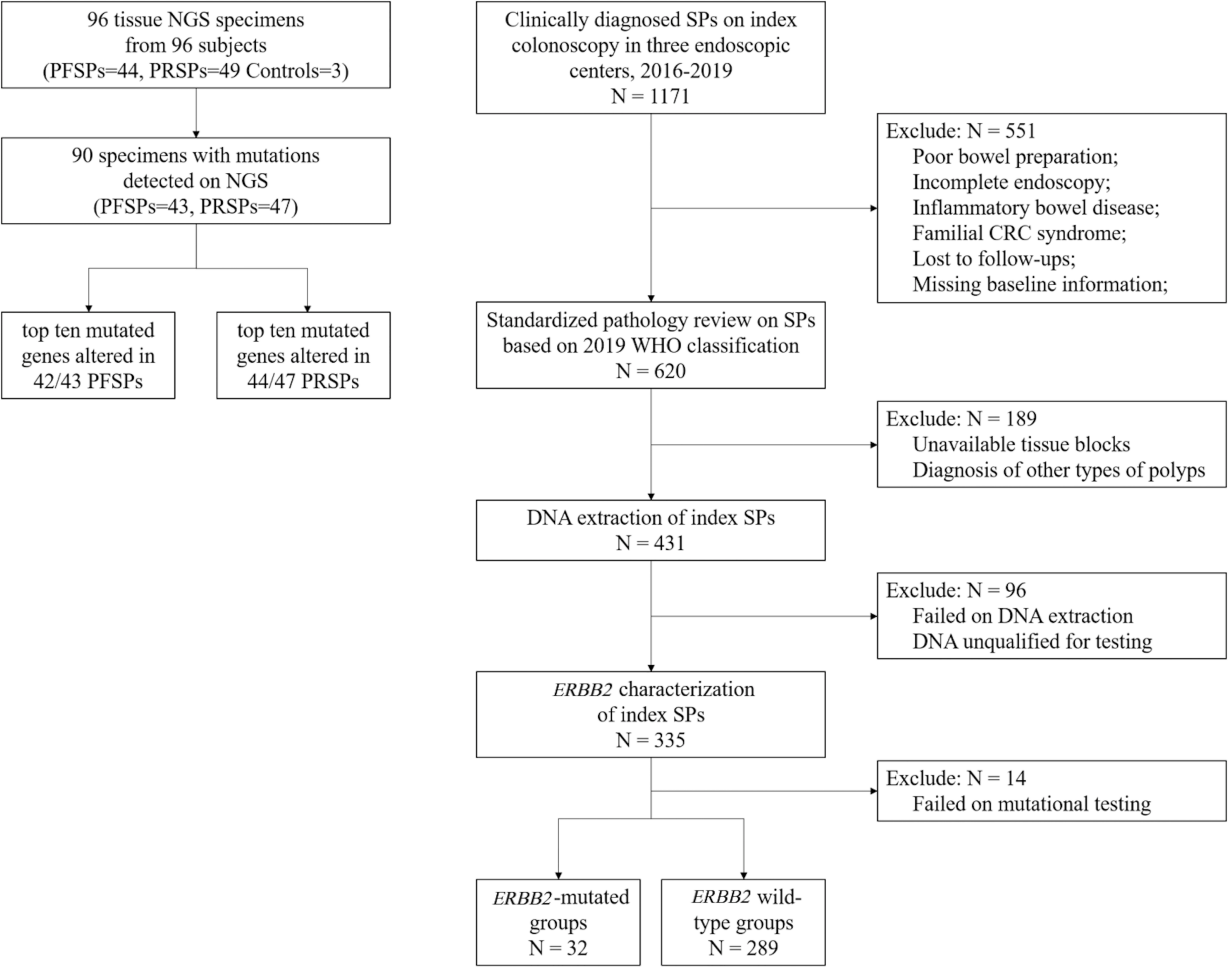
Cohort flowchart. NGS, next generation sequencing; PRSPs, polyp-relapsed serrated polyps; PFSPs, polyp-free serrated polyps; SPs, serrated polyps; CRC, colorectal cancer.

For the targeted NGS cohort, 96 candidates were enrolled at the Department of Gastroenterology and Hepatology, Ren-ji Hospital, Shanghai, between 2016 and 2019. In total, 93 colorectal SPs including 49 PRSPs and 44 PFSPs and 3 normal colon mucosae were retrieved from the tissue bank. A multicenter validation cohort was established longitudinally in Shanghai Renji Hospital, Chongqing Traditional Chinese Medicine Hospital and Sichuan Provincial Corps Hospital of Chinese People’s Armed Forces to confirm the relevant results. The overall schematic of the cohort selection is shown in **Figure 1**.

### Eligibility Criteria

The eligibility criteria of all the subjects in our study were as follows: age ≥18 years; received an index colonoscopy from 2016 to 2019 with a clinical diagnosis of SPs; fulfilled adequate bowel preparation and cecum reach. Subjects were ineligible if they had inflammatory bowel disease or familial CRC syndromes at index colonoscopy or were lost to follow-ups or baseline information. All parts of the colon were scrupulously examined, and all the polyps were completely removed on the index colonoscopy. All the subjects received surveillance colonoscopy annually with available demographics and clinicopathological medical data. The histology of all samples including index and recurrent polyps was reevaluated from pathology reports and biopsies by two pathologists to confirm the diagnosis according to the 2019 WHO classification. Written informed consent was obtained from each patient before the specimen collection. Approval of this study was achieved by the Ethics Committee of Renji Hospital, School of Medicine, Shanghai Jiao Tong University.

### Definitions

Since CRC was a relatively infrequent event among the subjects qualified with available medical records and regular surveillance in our study, we used risk of polyp recurrence as a surrogate marker. We defined PRSPs as the SPs with recurrence of polyps during surveillance colonoscopy, and PFSPs as the SPs free of polyps during surveillance colonoscopy. Index colonoscopy was referred to as a baseline colonoscopy for participants. Index polyps were the polyps diagnosed at index colonoscopy. We acknowledged a polyp recurrence when polyps were diagnosed during surveillance colonoscopy after the index colonoscopy. Recurrent polyps were classified into SPs (including HPs), non-advanced adenomas (NAs), or advanced neoplastic lesions (ANLs). ANLs included advanced adenomas (AAs) and cancers. AAs were considered as advanced adenomas that measure ≥10 mm in size, with villous or tubulovillous component, high-grade dysplasia, intramucosal carcinoma, or any combination thereof (14). Patients’ polyp-free intervals were calculated according to the time interval from the date of the index colonoscopy to the first diagnosis of polyp recurrence or until the date of last negative surveillance colonoscopy.

### DNA Extraction and Targeted Sequencing Analyses

Genomic DNA was extracted from 10-μm formalin-fixed, paraffin-embedded (FFPE) tissue samples using the QIAamp DNA FFPE Tissue Kit (Qiagen Inc., Chatsworth, CA, USA), following the manufacturer’s instructions. DNA quantity and quality were checked using Qubit dsDNA HS assay on the Qubit Fluorometer (Thermo Fisher Scientific, Waltham, MA). DNA libraries were constructed from 100 to 250 ng DNA samples using QIAseq Targeted DNA Human Colorectal Cancer Panel (#DHS-002Z, Qiagen Inc., Chatsworth, CA, USA) including 71 most commonly mutated genes in human CRCs. All DNA fragments were tagged with a unique molecular index (UMI; a 12-nucleotide random sequence).

Libraries were sequenced *via* the Illumina NextSeq instrument with 75~155-bp paired-end reads. Sequence reads were aligned to the hg19 human genome build. Sufficient sequencing quality was guaranteed from all samples with the 30× minimum total read depth. Variant detection, annotation, scoring, and further filtering were implemented by Biomedical Genomics Workbench version 5 (Qiagen, Valencia, CA, USA) with the gene panel-specific plugin QIAseq Targeted Panel Analysis.

The plugin used five different filters for removal of called variants. The confidence filter only retained variants that were not residing in the top 5% of the most exonically variable 100-base windows in healthy public genome database (four reference databases: Allele Frequency Community; 1000 Genomes Project; ExAC; NHLBI ESP exomes). Besides, the confidence filter removed all variants below a call quality of 20 and with a prevalence of 0.5% in the healthy population. The genetic analysis filter only kept variant UMI level allele fraction (VMF) that ranged from 1% to 45% for each tested region, described to be pathogenic and/or likely pathogenic or loss of function-associated, which causes frameshift, missense, etc. An R Bioconductor package, maftools, was used for integrative analysis of somatic variants (15). The ggplot2 package was applied to draw the distribution map of mutation.

### *ERBB2* Mutational Validation by Sanger Sequencing

DNA samples from the validation cohort were tested by *ERBB2* mutation status (R678Q, V842I) using Sanger sequencing. The two designed fragments of *ERBB2* domain mutations from the DNA of the patients were amplified by multiplex PCR using primers as follows: *ERBB2* R678Q forward (5’-GTTGGCATTCTGCTGGTCGT-3’) and reverse (5’-AGCAGTCTCCGCATCGTGTA-3’); *ERBB2* V842I forward (5’-GCTAGGATGGGGACTCTTGC-3’) and reverse (5’-CCCCCATCTGCATGGTACTC-3’). The obtained reaction products were confirmed successful amplification by electrophoresis and then subjected to direct sequencing and analyzed on a 3730xl DNA Analyzer (Applied Biosystems). Sequencing results were compared with the reference DNA sequence and were interpreted by two separate approaches to improve the mutation detection: electronically with a set threshold of 10% and by visual inspection of the electropherogram by two researchers using Sequencher 5.0 software.

### Statistical Analysis

R (Version 4.0.3) was used for statistical analysis. Continuous parameters expressed as mean ± SD were analyzed by one-way analysis of variance (ANOVA) or independent Student’s t-test, while comparison between categorial data was evaluated by chi-square test or Fisher’s exact test. Cox regression models were applied for the time-to-event outcome analysis. Kaplan–Meier curves were reported by log-rank test for the variants detected to assess the polyps-free probability. Hazard ratios (HRs) and 95% confidence intervals (CIs) were reported in Cox regression models, and P values from a likelihood ratio test less than 0.05 were considered statistically significant. All the P values were two-tailed.

## Results

### Patient Characteristics for the Targeted Next-Generation Sequencing

**Figure 2:**
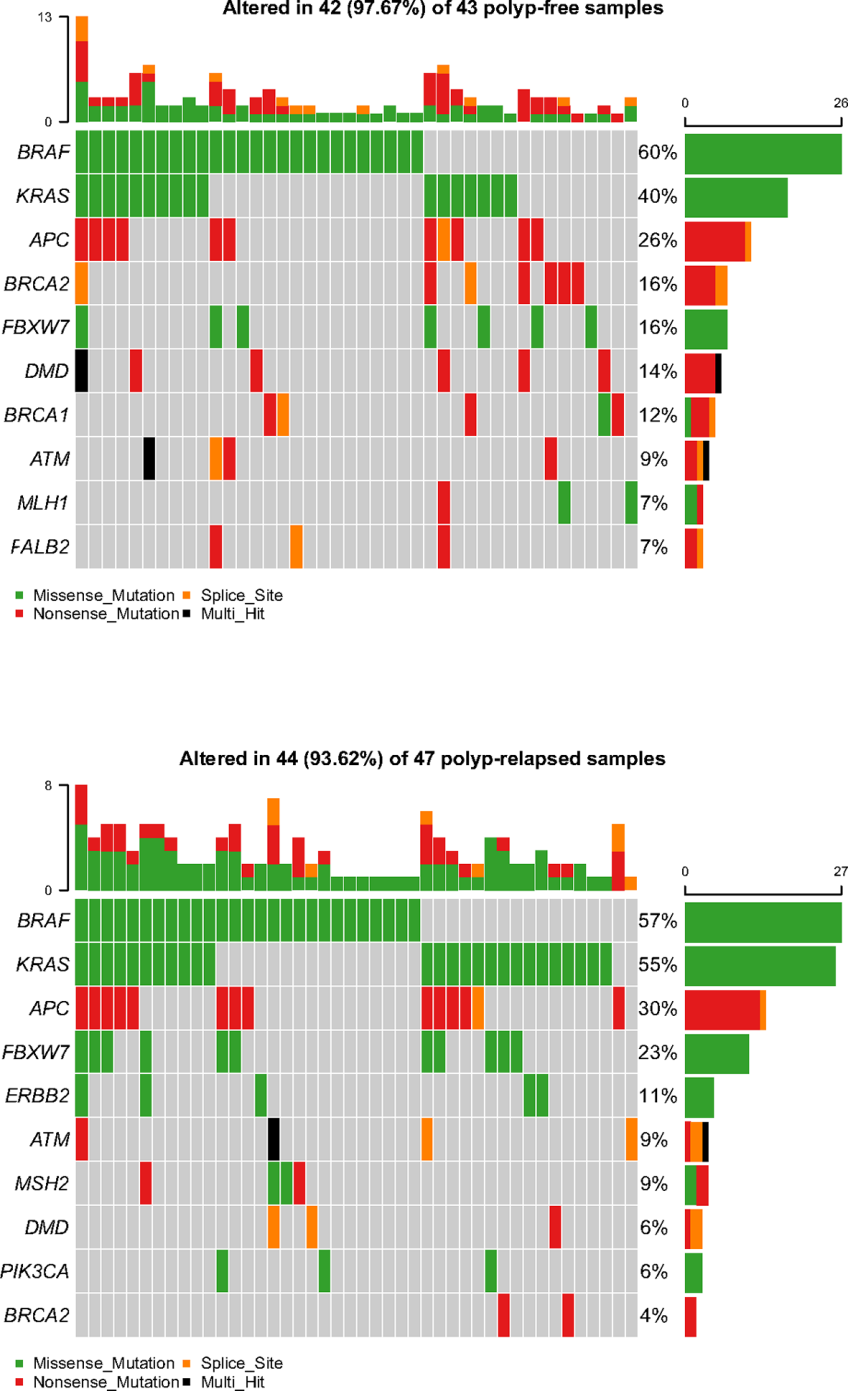
Representative spectrum of frequently mutated genes in polyp-relapsed and polyp-free colorectal serrated polyps after variant filtration.

The baseline information of the sequencing samples (n = 96) was listed in **Supplementary Table S1**. The ANNOVAR package was used to annotate the variants with all available public population information (16). The identified variants were classified as benign or pathogenic according to ClinVar database (17). In total, 90/96 samples (94%) (including 47 PRSPs and 43 PFSPs) displayed at least one pathogenic or likely pathogenic variant that was categorized as missense, nonsense mutation, or splice site according to the frequency-based analysis (**Figure 2**). The final filtered NGS cohort (n = 90) was listed in **Table 1**.

**Table 1:** Baseline characteristics of PRSPs and PFSPs after variant filtration (n = 90).

	PRSPs (n = 47)	PFSPs (n = 43)	P
Gender, n (%)			0.666
Men	23 (48.9)	23 (53.5)	
Women	24 (51.1)	20 (46.5)	
Age (years), mean (SD)	62.0 (10.5)	62.5 (9.7)	0.804
Polyp size (mm), mean (SD)	18.3 (13.8)	8.2 (6.5)	<0.001
Shape, n (%)			0.866
Pedunculated	17 (37.0)	13 (30.2)	
Flat	27 (58.7)	28 (65.1)	
Unknown	3 (4.3)	2 (4.7)	
Location, n (%)			0.571
Left colon	29 (61.7)	24 (55.8)	
Right colon	18 (38.3)	19 (44.2)	
Diagnosis, n (%)			<0.001
HPs	3 (6.4)	19 (44.2)	
SSLs	27 (57.4)	11 (25.6)	
TSAs	17 (36.2)	13 (30.2)	
Dysplasia, n (%)			0. 321
LGD	26 (55.3)	22 (51.2)	
HGD	6 (12.8)	2 (4.7)	
≥3 synchronic polyps on index colonoscopy, n (%)	19 (40.4)	19 (44.2)	0.718
≥1 synchronic ANL on index colonoscopy, n (%)	7 (14.9)	3 (7.0)	0.320
Colonoscopy polyp-free interval months, median (IQR)	25 (15)	30 (16)	0.006

PRSPs, polyp-relapsed serrated polyps; PFSPs, polyp-free serrated polyps; Left colon, defined as colon distal to splenic flexure; Right colon, defined as colon proximal to splenic flexure; HPs, hyperplastic polyps; SSLs, sessile serrated lesions; TSAs, traditional serrated adenoma; LGD, low-grade dysplasia; HGD, high-grade dysplasia; ANL, advanced neoplasia lesion defined as colorectal cancer or adenoma with size > 1cm or >75% tubulovillous component and/or high-grade dysplasia; IQR, interquartile range.

We compared clinical characteristics between PRSPs and PFSPs. There were no significant differences in patients’ gender and age. SPs in PRSPs were significantly larger than that in PFSPs (P < 0.001). No differences were detected for polyp shape or location or dysplasia grade. Higher percentages of SSLs and TSAs were observed in PRSPs compared to PFSPs with remarkable differences (P < 0.001). The presence of three or more synchronous polyps or at least one ANL on index colonoscopy was not statistically different in the two groups. During the median polyp-free period of 26 months, three individuals in total developed ANLs during the surveillance colonoscopy. Polyp-free intervals {median months [interquartile range (IQR)]} were remarkably longer in PFSPs than those in PRSPs [25 (15) vs. 30 (16), P = 0.006].

### Mutational Profile Analyses

**Figure 3:**
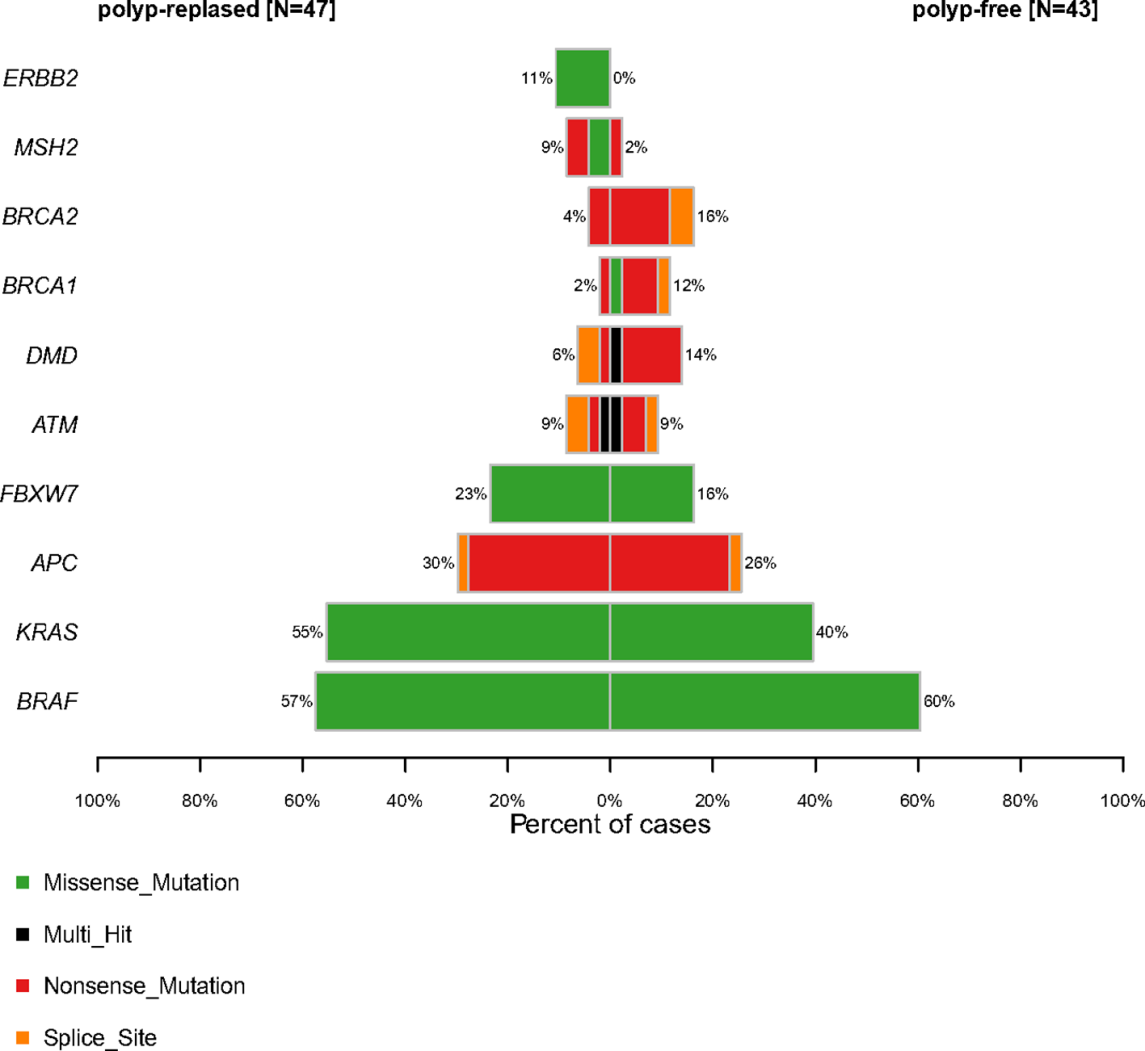
Comparative profiles of top 10 genomic alterations in polyp-relapsed and polyp-free colorectal serrated polyps.

Molecular profiling of PRSPs vs. PFSPs revealed that the three most common mutations were *BRAF* (57% vs. 60%), *KRAS* (55% vs. 40%), and *APC* (30% vs. 26%). *FBXW7* (23% vs.16%, P = 0.399), *MSH2* (9% vs. 2%, P = 0.413), and *ERBB2* (11% vs. 0%, P = 0.078) had a larger proportion in PRSPs than PFSPs, which might be recurrence-relevant genes. While *DMD* (6% vs. 14%, P = 0.399), *BRCA1* (2% vs. 12%, P = 0.167), and *BRCA2* (4% vs. 16%, P = 0.122) might be negatively correlated with recurrence (**Figure 3**).

### Kaplan–Meier Plot Analysis

**Figure 4:**
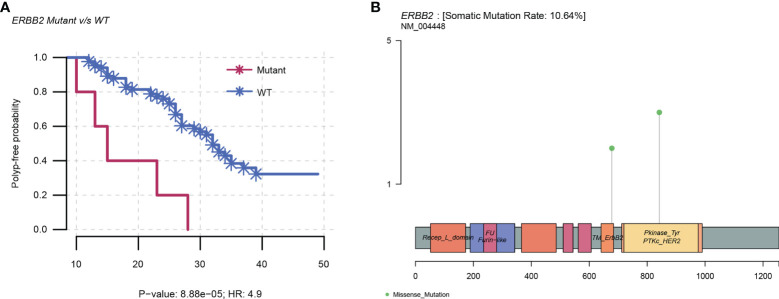
*ERBB2* mutations in the NGS cohort. **(A)** Kaplan–Meier plot showing an increased risk of polyp recurrence over time in the *ERBB2* mutants than in the wild types, with a median polyp-free interval of 15 months compared to 26 months (P < 0.001; HR = 4.9; 95% CI = 1.9–12.8). **(B)** Linear structure of *ERBB2* illustrating the region and frequency of the variants (R678Q and V842I). WT, wild type; HR, hazard ratio.

The top 10 mutated genes in the NGS cohort were all assessed for polyp-free intervals by Kaplan–Meier plot analysis (n = 90). The findings illustrated that only *ERBB2* mutations (n = 5) displayed a significant correlation with polyp recurrence, displaying a shorter median polyp-free interval of 15 months compared to 26 months than the wild types (n = 85) (P < 0.001; HR = 4.9; 95% CI = 1.9–12.8). Among the *ERBB2* somatic point mutations in five samples, three were in the protein kinase domain (V842I) and two were non-activating mutations (R678Q) (18) (**Supplementary Table S2** and **Figure 4**).

### Baseline Information for the Validation Cohort

The clinical relevance of *ERBB2* genes in serrated lesions with risks of polyp recurrence has not been documented yet. To further validate the presence of *ERBB2* mutations in SPs as a target of interest, we developed a separate validation cohort of 321 SPs from 308 patients recruited from three endoscopic centers (263 from Shanghai Ren-ji Hospital, 58 from the others). All the samples were screened for *ERBB2* mutation status (R678Q and V842I). The characteristics were shown in **Table 2**, with no statistically significant difference found among the 3 centers in baseline information and mutation frequencies (**Supplementary Table S3**).

**Table 2:** Baseline characteristics of the validation cohort of SPs according to findings on index colonoscopy (total n = 321).

	*ERBB2*-mutated SPs (n = 32)	Wild-type SPs (n = 289)	P
Gender, n (%)			0.474
Men	24 (75.0)	199 (68.9)	
Women	8 (25.0)	90 (31.1)	
Age, years, mean (SD)	58.3 (9.8)	58.6 (10.4)	0.863
Polyp size (mm), mean (SD)	9.1 (11.8)	7.1 (6.2)	0.033
Diagnosis, n (%)			0.019
HPs	19 (59.4)	201 (69.6)	
SSLs	7 (21.9)	72 (24.9)	
TSAs	6 (18.8)	16 (5.5)	
Location, n (%)			0.046
Left colon	10 (31.3)	144 (49.8)	
Right colon	22 (68.8)	145 (50.2)	
Dysplasia, n (%)			0.210
LGD	7 (21.9)	40 (13.8)	
HGD	1 (3.1)	4 (1.4)	
Colonoscopy polyp-free interval months, median (IQR)	26 (15)	32 (18)	0.008
Classifications of recurrent polyps during surveillance, n (%)			<0.001
SPs	15 (46.9)	46 (15.9)	
NAs	7 (21.9)	30 (10.4)	
ANLs	4 (12.5)	7 (2.4)	

Left colon, colon distal to splenic flexure; Right colon, colon proximal to splenic flexure; HPs, hyperplastic polyps; SSLs, sessile serrated lesions; TSAs, traditional serrated adenoma; LGD, low-grade dysplasia; HGD, high-grade dysplasia; SPs, serrated polyps; ANLs, advanced neoplasia lesions defined as colorectal cancer or adenoma with size > 1cm or >75% tubulovillous component and/or high-grade dysplasia; NAs, non-advanced adenomas; SD, standard deviation.

*ERBB2* mutations were detected in 32/321 SPs (10.0%), with 18/32 R678Q (56.3%) and 14/32 V842I (43.7%) mutants. There were no differences in terms of gender and age between *ERBB2*-mutated and wild-type patients. The *ERBB2*-mutated group tended to be more right-sided (68.8% vs. 50.2%, P = 0.046) and significantly larger than the wild-type group (9.1 ± 11.8 vs. 7.1 ± 6.2, P = 0.033). TSAs were more present in the *ERBB2*-mutated group (18.8%) than in the wild-type group (5.5%).

### Clinical Relevance of *ERBB2* Mutations in the Validation Cohort

**Figure 5:**
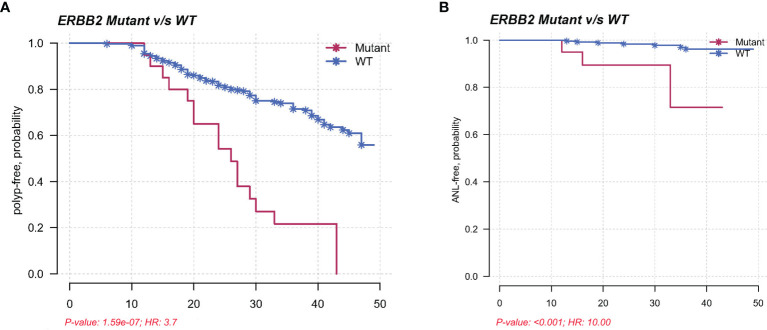
**(A)** Kaplan–Meier plot demonstrating a shorter polyp-free duration in the *ERBB2* mutants than in the wild types in a validation cohort, with a median interval of 26 months compared to 32 months (P < 0.001; HR = 3.7; 95% CI = 2.3–6.0). **(B)** Kaplan–Meier plot showing reduced ANL-free intervals in *ERBB2*-mutated SPs in a validation cohort (P < 0.001; HR = 10.0; 95% CI = 2.7–36.9).

In the validation cohort, Kaplan–Meier method and multivariate Cox regression were used to assess the association between *ERBB2* mutations and polyp recurrence. Cox regression analysis showed that age, pathology of SSLs, and *ERBB2* mutations independently predicted polyp recurrence. *ERBB2*-mutated SPs displayed increased risks of polyp recurrence, with a median polyp-free interval of 26 months compared to 32 months in wild-type SPs (P < 0.001; HR = 3.7; 95% CI = 2.3–6.0). *ERBB2* mutations were further associated with reduced ANL-free intervals in SPs with statistical significance (P < 0.001; HR = 10.0; 95% CI = 2.7–36.9) (**Figure 5**). *ERBB2* mutants displayed increased risks of polyp recurrence in Shanghai Renji center (P < 0.001), while no mutational factor was associated with polyp recurrence in patients from Chongqing TCM or Sichuan centers possibly due to the small sample size (**Supplementary Figure S1**).

In general, it is consistently suggested that patients with SSLs and TSAs yielding increased risks of metachronous ANLs compared with persons without polyps should have more aggressive surveillance. To distinguish patients impaired from pathology of SSLs and TSAs, we stratified the patients according to histological diagnosis. The Kaplan–Meier curves illustrated that patients with *ERBB2*-mutated HPs and SSLs had strikingly shorter polyp-free intervals compared with corresponding wild-type groups, respectively (P < 0.001; P = 0.036), while in patients with TSAs, no difference in recurrent outcome was observed (**Supplementary Figure S2**).

## Discussion

It has not been widely appreciated until 2003–2005 that distinct from conventional sequence, SPs are integrated into clinical practice as malignant precursors of CRCs through a serrated tumorigenic pathway. Clinical surveillance recommendations for SP patients on colonoscopy are difficult to set up uniformly on account of unknown underlying genetic mechanisms behind risks of subsequent CRCs (19–22). Controversy exists around the effectiveness of colonoscopy even based on the foremost guidelines, as unexpected interval cancers are sporadically developed within serial surveillance colonoscopies (23). Research regarding the remarkable resemblance of interval cancers to SPs in right-sided preference and epigenetic features such as MSI and CIMP (24, 25) has aroused extensive concern on identification of high-risk SPs in the clinic. Though various studies have documented generally accepted features of high-risk serrated lesions such as SSLs with size larger than 10 mm or SLs harboring dysplasia including TSAs that require intensive surveillance colonoscopy (5–7, 19), they are based on limited evidence. A recent study firstly provided longitudinal evidence on molecular markers (including *BRAF* V600E, CIMP, and *MLH1* methylation) of SPs, suggesting that epigenetic defect of *MLH1* methylation was in relation to subsequent advanced neoplasms (26). However, somatic mutations have not been screened yet.

In the present study, we firstly investigated the genetic alterations of SPs in relation to polyp recurrence by NGS. The mutational profile analyses demonstrated pathogenic variants in genes as previously reported, such as *BRAF* V600E and *KRAS* codon12/13 but also illustrated genes less recorded such as *FBXW7*, *ATM*, and *DMD*. This work provided several interesting findings. First, *BRAF*, *KRAS*, and *APC* were the key drivers of serrated tumorigenesis but not the useful markers to identify high-risk SPs. Second, higher prevalence of *FBXW7*, *MSH2*, and *ERBB2* mutants in PRSPs rather than PFSPs might suggest their association with higher risks of developing colorectal polyps. Third, lower incidences of *DMD*, *BRCA1*, and *BRCA2* mutants in PRSPs rather than PFSPs delineated the unlikelihood of their contribution to interval cancer. Finally, *ERBB2* mutants demonstrated a significant relationship with colorectal advanced neoplasm recurrence, implicating its value as an important marker of high-risk SPs.

In line with previous studies, our findings recognized *BRAF* V600E or *KRAS* mutations as the triggering event of the serrated pathway (27). The overall rate of *BRAF* V600E (53/90, 59%) was within the range reported in the existing literatures from 50% to 83% (28, 29). The high level of mutant *BRAF* in SPs is becoming growingly relevant as a poor prognostic factor (30). However, our result indicated that *BRAF* V600E was not related to the future risks of neoplasms, consistent with the result of Hua et al. (26). The prevalence of *KRAS* mutations is discrete, ranging from 15% to 75% in colorectal polyps in previous studies (31, 32). The frequency of *KRAS* mutations in our NGS analysis was 48% (43/90). Interestingly, *APC* was the third most mutated gene among the SPs. *APC* gene typically acts as a driver gene in adenoma-to-carcinoma sequence through aberrant *Wnt* signaling (33). The potential role of *APC* mutations in the serrated pathway remains controversial. It is generally believed that Wnt pathway of SPs is mainly activated by a number of alternative mechanisms such as *PTPRK-RSPO3* fusions and *RNF43* mutations other than *APC* (28, 34). Although recently, research is suggesting that *APC* mutations are likely the main pathogenic reason for *WNT* signaling activation in serrated pathway based on their high frequency (35, 36).

*ERBB2* is a member of the ErbB receptor tyrosine kinase (RTK) family that transduces downstream signaling pathway, such as Phosphatidylinositol-4,5-bisphosphate 3-kinase (PI3K)/protein kinase B (AKT) axis through the active form of homodimer or heterodimer complexes with other RTKs (37). *ERBB2* overexpression occurs in many kinds of human cancers such as breast and ovarian cancers (38), and its association with poor prognosis in these cancers has been widely proven (39, 40). Moreover, the use of NGS has revealed the presence of *ERBB2* sequence mutations in human tumors over recent 10 years. GENIE consortium data published in 2017 have exhibited that *ERBB2* is altered in 4.69% of 2,081 CRC patients with R678Q and V842I present in 0.48% and 0.38% of all CRC patients, respectively (41). Given the nearly 5% frequency of *ERBB2* alterations in CRCs based on the current literatures, *ERBB2* becomes a promising target in anti-*ERBB2*-targeted therapies and prognoses. Whereas the presence of *ERBB2* point mutations in colorectal serrated precursors has not been published before. Our findings have yielded important insights into the functional consequences of *ERBB2* mutants. In our study, *ERBB2* was mutated in 5.56% of 90 NGS subjects, and it was altered in 9.97% of 321 validation subjects with no statistical differences. Given the current identification of *ERBB2* mutations in high-risk SPs, it could help shed some light on better understanding of polyp development and recurrence and could mark the significance that *ERBB2* mutations may serve as an independent biomarker for guidance of clinical surveillance strategy. Patients with *ERBB2* mutation-positive SPs may be recommended to receive more aggressive follow-up colonoscopy.

Our study has several strengths. First, SPs in this study are reclassified both historically and clinically. The historical reclassification was based on the newest 2019 WHO definitions by two independent pathologists, while the clinical subgroups were defined according to the follow-up outcomes. Second, this is the first cohort study exploring the correlation between high-risk genomic aberrations of SPs and recurrent outcome over time by using a targeted colorectal NGS panel. Prior literature only identified several endoscopic, histologic, or epigenetic features of serrated precursors that were strongly related to subsequent high-risk adenomas or CRCs identified during surveillance colonoscopy. Finally, the correlation between the *ERBB2* mutational targets and colorectal polyp recurrence is first identified in the NGS cohort and was ulteriorly validated in a multicenter cohort. *ERBB2* mutations are qualified as a clinically relevant biomarker to predict risks of future ANLs that have HRs >3.

This study has likewise some limitations. The retrospective study contained only a small number of SPs, and the follow-up duration was relatively short. Sanger sequencing platform had the limitation for reliable detection that required more than 10% fraction of mutated allele fraction. A sensitive digital PCR-based *ERBB2* assay should be established in our future work to accurately evaluate its performance. The exploration of relevant factors of recurrence was limited in genomic features, while epigenetic characteristics of SPs were not dug out. Hence, the prognostic application of *ERBB2* mutants in serrated pathway should be confirmed prospectively and extensively in the future.

In summary, for the first time, we have identified distinct genomic features of SPs in relation to subsequent polyp recurrence. This is also the first study detecting *ERBB2* mutants in SPs. The clinical relevance of *ERBB2* mutants with higher risks of subsequent colorectal neoplasms suggests their prospects as molecular markers for high-risk SP identification.

## Data Availability Statement

The original contributions presented in the study are publicly available. These data can be found here: https://ngdc.cncb.ac.cn/gsa-human, PRJCA007048 and GSA-Human: HRA001502.

## Ethics Statement

The studies involving human participants were reviewed and approved by the Ethics Committee of the Renji Hospital, School of Medicine, Shanghai Jiao Tong University. The patients/participants provided their written informed consent to participate in this study.

## Author Contributions

Guarantor of the paper: X-BL and H-MC. Specific author contributions: Q-WW and X-YW performed the experiments, analyzed data, and wrote the original draft. H-YC revised the article. Q-WZ, J-NC, and Y-JZ collected clinical and pathological information. Z-RT and R-LW provided tissue specimens. X-BL and H-MC conceived and supervised the study. All the authors revised and approved the final article.

## Funding

The project described was supported by a funding grant from the National Natural Science Foundation of China (NSFC) (General Program: 81772519) and by a grant from the Excellent Youth Program of Shanghai Municipal Commission of Health and Family Planning (2018YQ29). There was no influence from the NSFC or Shanghai Municipal Commission of Health and Family Planning on study design or conduct, data collection and management, analysis, interpretation, preparation and review or approval of the article.

## Conflict of Interest

The authors declare that the research was conducted in the absence of any commercial or financial relationships that could be construed as a potential conflict of interest.

## Publisher’s Note

All claims expressed in this article are solely those of the authors and do not necessarily represent those of their affiliated organizations, or those of the publisher, the editors and the reviewers. Any product that may be evaluated in this article, or claim that may be made by its manufacturer, is not guaranteed or endorsed by the publisher.

## Supplementary Material

Supplementary Figure 1
**(A)** Kaplan-Meier plot showing no mutational factor associated with polyp recurrence in patients from Chongqing TCM (P = 0.086). **(B)** *ERBB2* mutants displaying increased risks of polyp recurrence, with a median polyp-free interval of 27 months compared to 40 months in wild types from Shanghai Renji center (P<0.001).

Supplementary Figure 2 Kaplan-Meier plot demonstrating a shorter polyp-free duration in the *ERBB2* mutated HPs **(A)**/SSLs **(B)** than in the corresponding wild-type groups in the validation cohort, with a median interval of 27/23 months compared to 43/34 months (P < 0.001; P = 0.036). **(C)** Kaplan-Meier plot showing no difference in recurrent outcome in validation cohort of TSAs (P=0.345).

Supplementary Table 1 Baseline characteristics of FFPE sections for sequencing (total n=96).

Supplementary Table 2 Summary of clinicopathological features in the NGS cases with *ERBB2* mutants (n=5).

Supplementary Table 3 Baseline characteristics of the validation cohort of SPs classified by clinical centers (total n=321).
