# Supplementary figures and images for: ERBB2 Mutations as Potential Predictors for Recurrence in Colorectal Serrated Polyps by Targeted Next-Generation Sequencing
Source: Front Oncol. 2022 Mar 23;12:769709. doi: 10.3389/fonc.2022.769709 (PMC8984468; doi:10.3389/fonc.2022.769709)

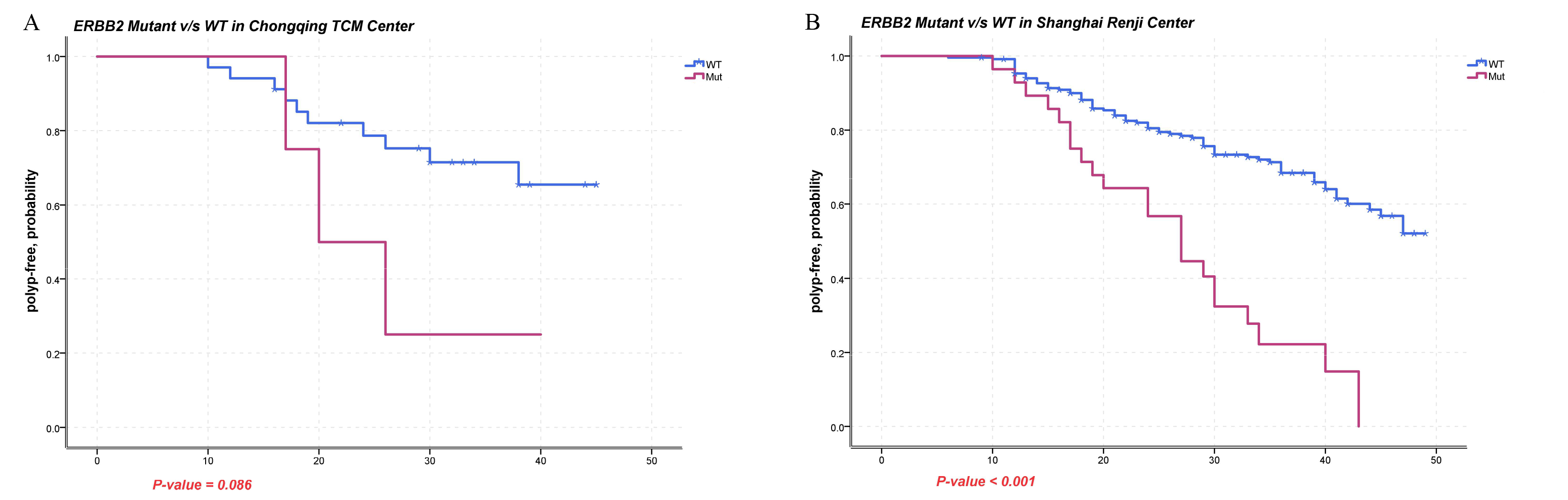

Supplement: Supplementary Figure 1 — (A) Kaplan-Meier plot showing no mutational factor associated with polyp recurrence in patients from Chongqing TCM (P = 0.086). (B) ERBB2 mutants displaying increased risks of polyp recurrence, with a median polyp-free interval of 27 months compared to 40 months in wild types from Shanghai Renji center (P<0.001). [file Image_1.tif]

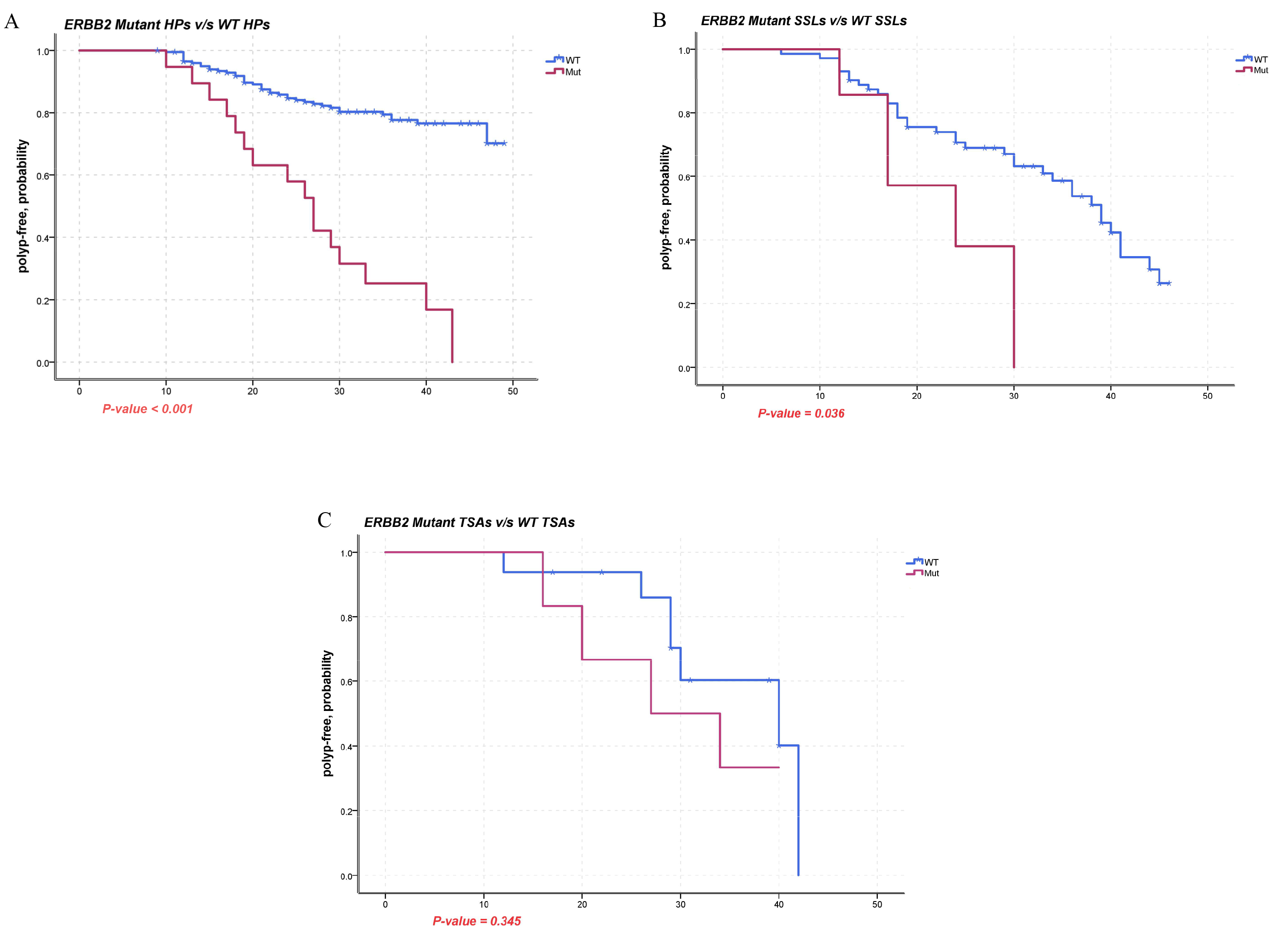

Supplement: Supplementary Figure 2 — Kaplan-Meier plot demonstrating a shorter polyp-free duration in the ERBB2 mutated HPs (A)/SSLs (B) than in the corresponding wild-type groups in the validation cohort, with a median interval of 27/23 months compared to 43/34 months (P < 0.001; P = 0.036). (C) Kaplan-Meier plot showing no difference in recurrent outcome in validation cohort of TSAs (P=0.345). [file Image_2.tif]
