# Supplementary material for: ERBB2 Mutations as Potential Predictors for Recurrence in Colorectal Serrated Polyps by Targeted Next-Generation Sequencing
Source: Front Oncol. 2022 Mar 23;12:769709. doi: 10.3389/fonc.2022.769709 (PMC8984468; doi:10.3389/fonc.2022.769709)
Supplement: Supplementary Table 1 — Baseline characteristics of FFPE sections for sequencing (total n=96). [file Table_1.docx]

| **Supplemental Table 1. Baseline characteristics of FFPE sections for sequencing (total n=96)** | | | | |
| --- | --- | --- | --- | --- |
|  | **Controls**  **(n=3)** | **Polyp-relapsed SPs**  **(n=49)** | **Polyp-free SPs**  **(n=44)** | **P value**  **PRSPs vs. PFSPs** |
| **Gender, n (%)** |  |  |  | **0.904** |
| **Male** | **2 (66.7)** | **25 (51.0)** | **23 (52.3)** |  |
| **Female** | **1 (33.3)** | **24 (49.0)** | **21 (47.7)** |  |
| **Age, years, mean (s.d.)** | **64 (5.0)** | **62.3 (10.5)** | **62.5 (9.6)** | **0.933** |
| **Polyp size (mm), mean (s.d.)** | **/** | **18.0 (13.5)** | **8.2 (6.4)** | **<0.001** |
| **Shape, n (%)** |  |  |  | **0.831** |
| **Pedunculated** | **/** | **17 (34.7)** | **13 (29.5)** |  |
| **Flat** | **/** | **29 (59.2)** | **29 (66.0)** |  |
| **Unknown** | **/** | **3 (6.1)** | **2 (4.5)** |  |
| **Location, n (%)** |  |  |  | **0.817** |
| **Left colon** | **1 (33.3)** | **29 (59.2)** | **25 (56.8)** |  |
| **Right colon** | **2 (66.7)** | **20 (40.8)** | **19 (43.2)** |  |
| **Diagnosis, n (%)** |  |  |  | **<0.001** |
| **HPs** | **/** | **4 (8.2)** | **20 (45.5)** |  |
| **SSLs** | **/** | **28 (57.1)** | **11 (25.0)** |  |
| **TSAs** | **/** | **17 (34.7)** | **13 (29.5)** |  |
| **Dysplasia, n (%)** |  |  |  | **0. 212** |
| **LGD** | **/** | **26 (53.1)** | **22 (50.0)** |  |
| **HGD** | **/** | **7 (14.3)** | **2 (4.5)** |  |
| **≥3 synchronic polyps on index colonoscopy, n (%)** | **0 (0)** | **20 (40.8)** | **19 (43.2)** | **0.836** |
| **≥1 synchronic ANL or carcinoma on index colonoscopy, n (%)** | **0 (0)** | **7 (14.3)** | **3 (6.8)** | **0.409** |
| **Colonoscopy polyp-free interval months, median (IQR)** | **33 (11.5)** | **24 (14)** | **30 (15.75)** | **0.002** |

**FFPE, formalin-fixed paraffin-embedded; PRSPs, polyp-relapsed serrated polyps; PFSPs, polyp-free serrated polyps; Left colon, defined as colon distal to splenic flexure; Right colon, defined as colon proximal to splenic flexure; HPs, hyperplastic polyps; SSLs, sessile serrated lesions; TSAs, traditional serrated adenoma; LGD, low-grade dysplasia; HGD, high-grade dysplasia; ANL, advanced neoplastic lesion, defined as colorectal cancers or those adenomas with size > 1cm or >75% tubulovillous component and/or high-grade dysplasia.**
