# Supplementary material for: ERBB2 Mutations as Potential Predictors for Recurrence in Colorectal Serrated Polyps by Targeted Next-Generation Sequencing
Source: Front Oncol. 2022 Mar 23;12:769709. doi: 10.3389/fonc.2022.769709 (PMC8984468; doi:10.3389/fonc.2022.769709)
Supplement: Supplementary Table 2 — Summary of clinicopathological features in the NGS cases with ERBB2 mutants (n=5). [file Table_2.docx]

| **Supplemental Table 2. Summary of clinicopathological features in the NGS cases with ERBB2 mutants (n=5)** | | | | | | | | |
| --- | --- | --- | --- | --- | --- | --- | --- | --- |
| **Case No.** | **Age** | **Gender**  **(M/F)** | **Location**  **(R/L)** | **Size (mm)** | **Histological type** | **ERBB2 mutant** | **Follow-up duration months** | **Recurrent polyp types** |
| **1** | **67** | **F** | **R** | **18** | **HP** | **V842I** | **23** | **SPs** |
| **2** | **49** | **M** | **R** | **21** | **SSL-LGD** | **R678Q** | **13** | **NAs** |
| **3** | **31** | **M** | **R** | **20** | **SSL-HGD** | **R678Q** | **28** | **NAs** |
| **4** | **42** | **M** | **R** | **3** | **TSA** | **V842I** | **10** | **NAs** |
| **5** | **66** | **F** | **L** | **12** | **TSA** | **V842I** | **15** | **SPs** |

**M, male; F, female; R, right colon, defined as colon proximal to splenic flexure; L, left colon, defined as colon distal to splenic flexure; HPs, hyperplastic polyps; SSLs, sessile serrated lesions; TSAs, traditional serrated adenoma; LGD, low-grade dysplasia; HGD, high-grade dysplasia; SPs, serrated polyps; NAs, nonadvanced adenomas.**
