# Supplementary material for: ERBB2 Mutations as Potential Predictors for Recurrence in Colorectal Serrated Polyps by Targeted Next-Generation Sequencing
Source: Front Oncol. 2022 Mar 23;12:769709. doi: 10.3389/fonc.2022.769709 (PMC8984468; doi:10.3389/fonc.2022.769709)
Supplement: Supplementary Table 3 — Baseline characteristics of the validation cohort of SPs classified by clinical centers (total n=321). [file Table_3.docx]

| **Supplemental Table 3. Baseline characteristics of the validation cohort of SPs classified by clinical centers (total n=321)** | | | | |
| --- | --- | --- | --- | --- |
|  | **Shanghai Renji**  **( n = 263 )** | **Chongqing TCM**  **( n = 38 )** | **Sichuan**  **( n = 20 )** | ***P* value** |
| **Gender, n (%)** |  |  |  | **0.108** |
| **Male** | **184 (70.0)** | **29 (76.3)** | **10 (50.0)** |  |
| **Female** | **79 (30.0)** | **9 (23.7)** | **10 (50.0)** |  |
| **Age, years, mean (s.d.)** | **58.3 (10.5)** | **60.3 (8.9)** | **59.5 (9.4)** | **0.438** |
| **Polyp size (mm), mean (s.d.)** | **7.4 (7.2)** | **7.45 (6.7)** | **5.8 (2.3)** | **0.598** |
| **Diagnosis, n (%)** |  |  |  | **0.131** |
| **HPs** | **186 (70.7)** | **22 (57.9)** | **12 (60.0)** |  |
| **SSLs** | **62 (23.6)** | **10 (26.3)** | **7 (35.0)** |  |
| **TSAs** | **15 (5.7)** | **6 (15.8)** | **1 (5.0)** |  |
| **Location, n (%)** |  |  |  | **0.699** |
| **Left colon** | **134 (51.0)** | **22 (57.9)** | **11 (55.0)** |  |
| **Right colon** | **129 (49.0)** | **16 (42.1)** | **9 (45.0)** |  |
| **ERBB2** |  |  |  | **0.369** |
| **Mutants** | **28 (10.6)** | **4 (10.5)** | **0 (0.0)** |  |
| **Wild types** | **235 (89.4)** | **34 (89.5)** | **20 (100.0)** |  |
| **Colonoscopy polyp-free interval months, median (IQR)** | **30 (11)** | **30 (10)** | **34 (8)** | **0.426** |

**HPs, hyperplastic polyps; SSLs, sessile serrated lesions; TSAs, traditional serrated adenoma; Left colon, defined as colon distal to splenic flexure; Right colon, defined as colon proximal to splenic flexure.**
